# A Case of Daptomycin-Induced Eosinophilic Pneumonia and Its Management Insights

**DOI:** 10.7759/cureus.83195

**Published:** 2025-04-29

**Authors:** Diaz Saez Yordanka, Nishant Allena, Swetha Doddi, Harish Patel, Neelanjana Pandey, Trupti Vakde

**Affiliations:** 1 Pulmonary and Critical Care Medicine, BronxCare Health System, Bronx, USA; 2 Internal Medicine, BronxCare Health System, Bronx, USA

**Keywords:** bronchoalveolar lavage, daptomycin-induced acute eosinophilic pneumonia, drug induce pneumonitis, peripheral eosinophilia, pulmonary infiltrates

## Abstract

Pulmonary infiltrates, arising from diverse etiologies such as infections, cardiac conditions, or parenchymal diseases, present a diagnostic challenge. Drug-induced pneumonitis, although less common, should be considered, especially when symptoms develop after medication initiation. This case report highlights a rare yet significant complication of antibiotic therapy, daptomycin-induced eosinophilic pneumonia (DIEP).

A 56-year-old male with a history of type 2 diabetes mellitus, hypertension, and renal insufficiency presented with pleuritic chest pain and a productive cough for two days. Chest X-ray and CT imaging revealed bilateral scattered airspace opacities and ground-glass opacities, suggesting pneumonia or pulmonary edema. Initially treated for healthcare-associated pneumonia, the patient's condition persisted despite therapy. His medical history included osteomyelitis treated with vancomycin, later switched to daptomycin. Two weeks after the switch, the patient developed new respiratory symptoms. A bronchoalveolar lavage (BAL) was performed to establish the diagnosis of eosinophilic pneumonia. BAL showed >25% eosinophils, confirming daptomycin-induced eosinophilic pneumonia. The antibiotic was discontinued, and prednisone 40 mg daily was initiated, leading to the resolution of symptoms.

Daptomycin, an antibiotic commonly used to treat gram-positive infections, can rarely cause eosinophilic pneumonia, a rare adverse reaction characterized by pleuritic chest pain, dyspnea, and diffuse ground-glass opacities on imaging. The mechanism remains unclear but may involve surfactant binding, leading to alveolar epithelial injury. Diagnosis is confirmed through BAL, with eosinophilia greater than 25%. Management consists of discontinuing daptomycin and initiating steroids if necessary. This case underscores the importance of early recognition and prompt discontinuation of the offending drug, along with the use of steroids in cases with severe symptoms. BAL is a key diagnostic tool in confirming drug-induced eosinophilic pneumonia.

In conclusion, daptomycin-induced eosinophilic pneumonia is a rare but significant complication requiring vigilance in patients treated with the drug. Early diagnosis and effective management are crucial for achieving favorable outcomes.

## Introduction

Pulmonary infiltrates can be a challenging diagnostic dilemma, presenting various differential diagnoses that clinicians must consider. These infiltrates may arise from multiple infectious etiologies, including bacterial, viral, and fungal pathogens, or may signify pulmonary congestion related to cardiac conditions. Additionally, rare causes, such as pulmonary parenchymal diseases or certain interstitial conditions, must not be overlooked.

Nosocomial pulmonary infections are particularly prevalent, ranking as the second most common type of hospital-acquired infection, underscoring the importance of vigilance in identifying these conditions in hospitalized patients. One lesser-known but critical etiology of pulmonary infiltrates is drug-induced pneumonitis. Although not as frequently encountered, it is vital to include this in the differential diagnosis, especially when patients present with respiratory symptoms after the initiation of medication therapy [1,2].

In this report, we present a unique case of antibiotic-related eosinophilic pneumonitis, highlighting the significance of early recognition in altering the management and improving patient outcomes. This case illustrates the need for heightened awareness among healthcare providers regarding the potential pulmonary adverse effects of commonly used antibiotics, which can significantly impact clinical decision-making and patient care.

## Case presentation

A 56-year-old male with a history of type 2 diabetes mellitus, hypertension, and renal insufficiency presented to the emergency department with pleuritic chest pain and a productive cough with brown-colored sputum for the past two days. He denied associated symptoms such as fever, shortness of breath, palpitations, nausea, or vomiting. On examination, he was well-nourished, alert, and oriented. Vital signs revealed a temperature of 99°F, a heart rate of 110 bpm, blood pressure of 147/98 mm Hg, and normal oxygen saturation of 100% on room air.

**Figure 1:**
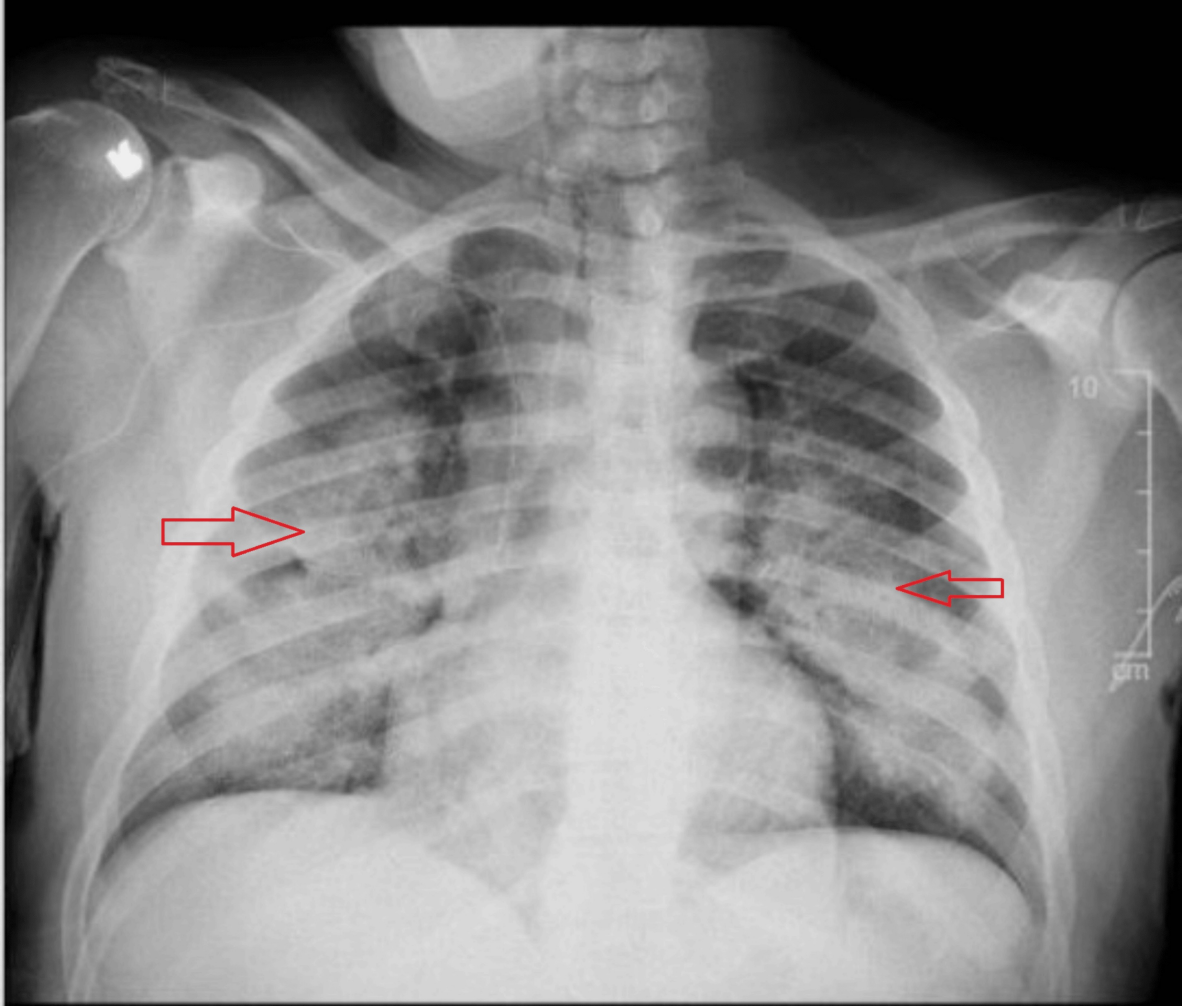
Chest X-ray showing bilateral scattered airspace opacities.

**Figure 2:**
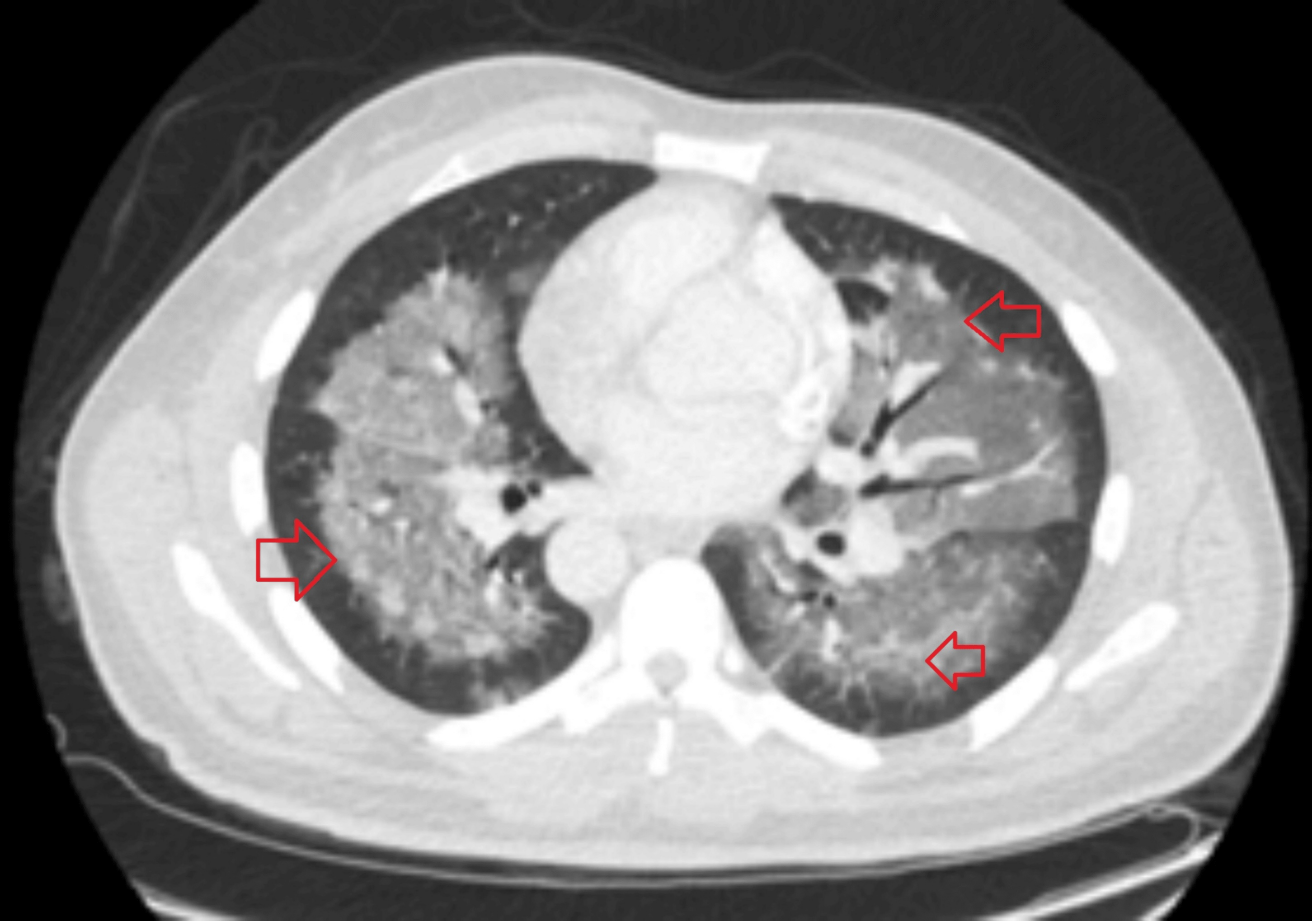
CT chest scan revealing confluent ground-glass opacities in centrilobular regions throughout both lungs.

Laboratory results indicated anemia (hemoglobin 8.9 g/dl), eosinophilia (13.6%), and a pro-B-type natriuretic peptide (BNP) level of 303 pg/ml (Table 1). Chest X-ray (Figure 1) and CT imaging revealed bilateral scattered airspace opacities and confluent ground-glass opacities, suggestive of pneumonia or pulmonary edema (Figure 2). The patient was initially treated for healthcare-associated pneumonia, and a pulmonary consultation was sought for further evaluation.

**Table 1:** Laboratory Values on Admission BNP: B-type natriuretic peptide

Parameter	Result	Reference Value
Hemoglobin	8.9 g/dL	12.0-16.0 g/dl
Eosinophils	13.60%	<5%
ProBNP	303	0-450 pg/mL

**Figure 3:**
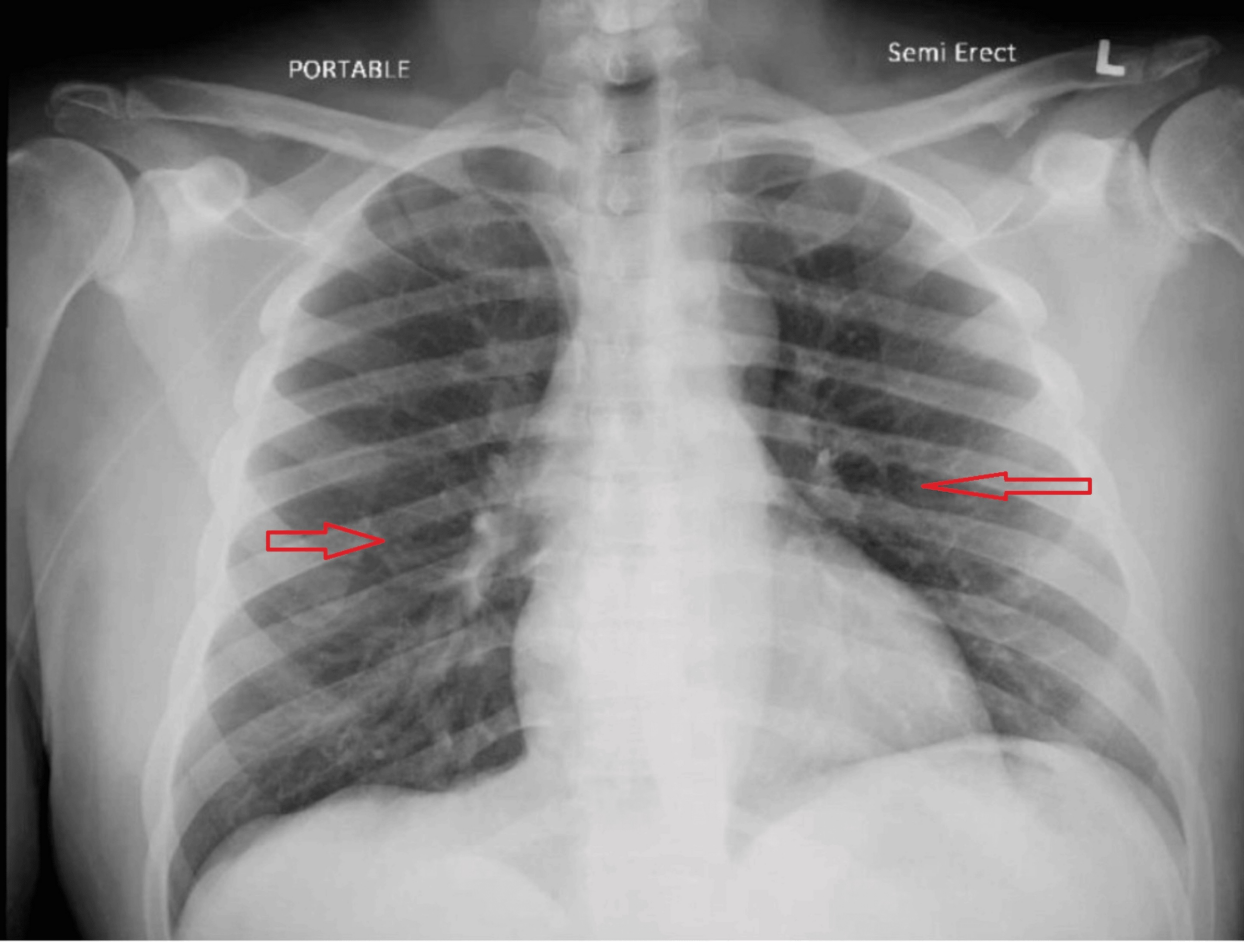
Prior chest X-ray showing no infiltrates or abnormalities.

The patient’s medical history included osteomyelitis of the left third toe, treated with vancomycin, later switched to daptomycin due to renal insufficiency. Two weeks into the daptomycin therapy, the patient developed new symptoms, prompting further investigations. The X-ray at his prior presentation had been normal, with no infiltrates noted (Figure 3).

A bronchoalveolar lavage (BAL) was planned to establish a diagnosis of eosinophilic pneumonia, which was crucial for justifying the initiation of corticosteroids in the presence of active osteomyelitis and for considering alternative antibiotics. BAL revealed >25% eosinophils (Table 2), indicative of eosinophilic pneumonia, with microbiological cultures from sputum, BAL, and blood negative for bacterial, viral, and fungal pathogens. Based on these clinical findings, a diagnosis of daptomycin-induced eosinophilic pneumonia (DIEP) was established. Daptomycin was discontinued, and the patient was initiated on prednisone 20 mg, with a resolution of symptoms. The patient was planned for outpatient follow-up.

**Table 2:** Bronchoaleolar lavage findings

Parameter	Results	Reference Value
Eosinophils Body Fluid	36.00%	1%

## Discussion

The increasing prevalence of bone and joint injections highlights the need for effective antibiotic prophylaxis and treatment in these cases. Infections following these procedures are commonly caused by organisms such as Staphylococcus, Enterococcus, and Corynebacterium, which may exhibit resistance to β-lactam antibiotics [3]. As a result, there has been an increase in the use of daptomycin due to its robust anti-gram-positive activity. Its utilization has become particularly common in managing skin and soft tissue infections [4]. However, achieving adequate bone penetration often necessitates the use of higher doses.

Daptomycin is often regarded as a safer alternative to vancomycin, with fewer adverse reactions reported [5]. One notable side effect associated with daptomycin therapy is the elevation of creatine phosphokinase, occurring in approximately 2% to 14% of patients, specifically when co-administered with statins [6]. While daptomycin's potential to cause eosinophilic pneumonia is reported less commonly, it is a vital complication to consider, especially if it leads to the discontinuation of treatment.

The exact mechanism underlying daptomycin-related eosinophilic pneumonia remains poorly understood. It has been suggested that daptomycin binds to calcium, facilitating its interaction with the cytoplasmic membrane and thereby increasing permeability [7]. There is speculation that daptomycin may bind to human surfactant, leading to accumulation in the alveolar space and subsequent epithelial injury [8]. According to FDA guidelines, daptomycin-associated eosinophilic pneumonia can be diagnosed when specific criteria are met, including concurrent exposure to daptomycin, respiratory symptoms, new infiltrates on imaging, and an elevated eosinophil count in BAL with >25% eosinophils, as well as improvement with drug withdrawal [9].

The exact incidence of daptomycin-related eosinophilic pneumonitis remains unclear. However, a study by Soldevila et al. revealed that approximately 4.8% of a cohort of 229 patients developed this condition. Several risk factors have been identified for developing daptomycin-related pneumonitis, including being over 70 years, a treatment duration exceeding two weeks, a higher cumulative dose of daptomycin, and an elevated Charlson Comorbidity Index. These factors should be carefully considered when evaluating patients treated with daptomycin to mitigate the risk of this adverse reaction [10]. Additionally, patients who develop peripheral eosinophilia following daptomycin therapy are also at increased risk for this adverse reaction [10]. These factors should be carefully considered when evaluating patients receiving daptomycin to mitigate the risk of eosinophilic pneumonitis effectively.

Radiologically, DIEP typically presents with bilateral ground-glass opacities and centrilobular nodules, predominantly located in the upper lobes. High-resolution CT plays a crucial role in differentiating DIEP from other potential causes, such as infections or interstitial lung diseases. In our patient’s case, the prominent centrilobular ground-glass opacities observed on imaging, although typically seen in diffuse alveolar damage (DAD), can also be indicative of DIEP.

Common symptoms associated with DIEP include pleuritic chest pain, dyspnea, cough, and occasionally fever, which generally manifest within two to four weeks post-initiation of daptomycin therapy. Peripheral eosinophilia is often seen in laboratory evaluations, and imaging frequently demonstrates diffuse ground-glass opacities. A BAL eosinophilia greater than 25% is considered confirmatory for the diagnosis of DIEP in the appropriate clinical context [10]. In this case, the patient's BAL eosinophilia and radiological findings were consistent with DIEP, reinforcing the diagnosis.

Given the potential for serious adverse reactions to daptomycin, patients experiencing daptomycin-related pneumonitis mustn't be rechallenged with the antibiotic. In cases where alternative antibiotic options are limited, establishing a definitive diagnosis becomes essential for appropriate management and ensuring patient safety. BAL plays a key role in confirming this diagnosis, as it provides evidence of an eosinophil count greater than 25%. Therefore, it is important to conduct BAL to establish the diagnosis accurately and guide clinical decision-making effectively.

The severity of daptomycin-related eosinophilic pneumonitis can vary among patients, but discontinuing daptomycin typically leads to the resolution of both symptoms and pulmonary infiltrates. Estimates suggest that approximately 15% of patients may not require any additional treatment beyond the cessation of the antibiotic, with clinical improvement often noticed within 24 hours of discontinuation. However, in a small subset of patients, particularly those presenting with hypoxia, steroids may be necessary if there is no improvement in the clinical condition. The optimal duration for spontaneous resolution remains unknown, but case series indicate that it may take up to 96 hours for symptoms to resolve fully [10].

## Conclusions

In conclusion, this case of daptomycin-induced eosinophilic pneumonia highlights the critical need for awareness and accurate diagnosis of drug-induced lung complications in patients receiving antibiotic therapy. The diverse clinical presentation of pulmonary infiltrates necessitates a heightened clinical suspicion for eosinophilic pneumonitis, particularly in those treated with daptomycin. BAL should be employed to confirm the diagnosis effectively. Early discontinuation of the offending antibiotic typically results in symptom resolution; however, steroid administration may often be required for optimal management. Importantly, patients who experience this reaction should never be rechallenged with daptomycin to avoid the recurrence of adverse effects
